# The Medical Department of the New Territorial Army

**Published:** 1907-10-26

**Authors:** 


### The Royal Army Medical Corps Section.

### THE MEDICAL DEPARTMENT OF THE NEW TERRITORIAL ARMY.

The official announcement that the Regulations for the Volunteers under the new Territorial Army Act will be issued in September will be viewed with a considerable amount of interest by those connected with the Medical Department of the Volunteer Force. For them the all-important question will naturally be what form the organisation of that medical department will take under the new scheme, and as the Act itself specifies but little under this head, much will depend upon the views of the Army Council as to the lines upon which the Medical Service shall be conducted. It is to be hoped that in the consideration they bestow upon this branch they will be guided by the experiences of recent campaigns, and that they will so frame the regulations that commanding officers of medical units may be enabled to strive towards an actual " war efficiency " rather than the securing of a " capitation grant."

There is much in the existing regulations which might well be amended in this respect. It is notorious that the Volunteer Force suffers, in common with the Regular Army, but to a much greater extent, from over-drilling and under-training; and in no department is this evil more noticeable than in the medical branch of the Service. The regulation which insists upon a prescribed course of infantry drill for stretcher-bearers and hospital orderlies is a very serious handicap on the attempt to secure a real efficiency. Under the present system much valuable time is absolutely wasted in useless preliminary drill which can have no practical bearing on the more serious work to follow. By the time the recruit is taught " how to walk and where to put his feet " his compulsory attendances are finished, and the training which should fit him for the real part he is called upon to play becomes optional, and is accordingly too frequently left alone. When the annual inspection time comes round valuable time is again lost in preparation for ceremonial drill and manœuvring, and when the inspecting officer pays attention only to the polishing of chin-straps and the pipe-claying of belts the man in the ranks is not to be blamed if his attention is concentrated more on these details than on First-aid or Sanitation. The fundamental idea should be efficient training in the special departments with the drill and ceremonial in the background, rather than the block-cut system which perpetuates the reverse condition of affairs. Just as the great aim of the rifleman should be to know how to shoot rather than how to put his toes at the " about turn," so the training of the officers and men of the Medical Service should be concentrated on the duties which they will be called upon to perform in time of war. If there are to be specialists the individuals must have the opportunities of training as specialists.

There are three main points, however, which call for consideration at the hands of the Army Council. The first of these, and, perhaps, the outstanding one at this juncture, is the organisation of the medical units both as regards officers and men. The evolution of the Royal Army Medical Corps, and the marked success which has attended the inclusion of the medical units of the Regular Army under one single organisation point the way to a satisfactory solution of the present difficulty. Under existing conditions the regimental system in the Volunteer Force is but a farce and the ambulance detachment of two men per company is non-existent. The regimental medical officer has no control over the men who are supposed to form his section, and the company officer has not only no inducement to detail men for ambulance work, but in view of his capitation grant just the reverse. As no capitation grant is secured for regimental ambulance training, the enthusiast must consider it as additional to his company drill and musketry.

To remedy such a condition of matters the medical officer must be enabled to enlist and train his men independently of the company officer, and the men must concentrate their training on ambulance work, each earning a capitation grant. Only thus can the men become efficient stretcher-bearers. The position of the officer himself must also be considered. It cannot be denied that many points connected with the old regimental system, which has been all but abolished in the Regular Army, had their advantages. But while Volunteer regimental officers are always a necessity, those officers should belong to a thoroughly organised medical department as in the home and Indian armies, from which they can be detached for regimental service. Such an arrangement would be much more satisfactory from an efficiency point of view not only to the medical branch of the Service as a whole, but to the officers individually, who would always feel that as part of such an organisation they would have effective support in the discharge of their duties. The position of the medical officer and his regimental bearers is a subject which is deserving of the serious consideration of the authorities.

The next point is the training of the officers and men, which has already been incidentally alluded to. The days of ceremonial drill with well-polished swords and pipe-clayed belts is, let us fervently hope, gone for evermore. But in its place we must have the means of training men in the duties for which they are detailed on paper. It is manifestly impossible to train a hospital orderly in the drill hall, and unless the medical private has hospital training he is simply on a level with the rifleman who has never handled a rifle. Several units have attempted to give their members this essential training, notably the Glasgow Companies of the Royal Army Medical Corps Volunteers (who send squads for week-end training during the winter months to Maryhill Station Hospital), the Warwick and Worcester Bearer Companies (who have had instruction in the Birmingham Hospital), and the London Companies, Royal Army Medical Corps Volunteers (who have secured training in one of the London hospitals). But this is only the fringe of the matter. Stretcherbearers can be trained in a few hours to load and carry stretchers, but the man who cannot be thus hurriedly instructed is the hospital orderly or nurse who is most in demand when the stress of war comes. Every medical orderly should have an opportunity of obtaining practical instruction in ward work, and only when he has so qualified himself should he be regarded as efficient. Several medical units have been accustomed to obtain such training during their annual camp at Netley Hospital, where a very thorough course of practical instruction is arranged. But during the past summer this arrangement was negatived for Scotland with disastrous results, and the week at camp consequently shorn of all its advantage. It is to be hoped that no such shortsighted policy will be possible under the new scheme.

Lastly, there is the question of equipment. To train the hospital orderly without field hospital equipment, such as he will utilise on service, is like training artillerymen without guns. On paper the medical units of the Volunteer force have been able in recent years to staff a certain number of field hospitals, yet not one of those units had ever been given the opportunity of recognising a field hospital when they saw one. The new field ambulance is now organised and is doubtless a vast improvement from every point of view, but unless its equipment is provided for training it would be futile to expect efficiency. These points require the earnest attention of the authorities, who so much desire to see the Volunteer force on a proper basis.

